# Supplementary figures and images for: A Narrative Review of the History of Burn-Related Depression and Stress Reactions
Source: Medicina (Kaunas). 2022 Oct 5;58(10):1395. doi: 10.3390/medicina58101395 (PMC9609573; doi:10.3390/medicina58101395)

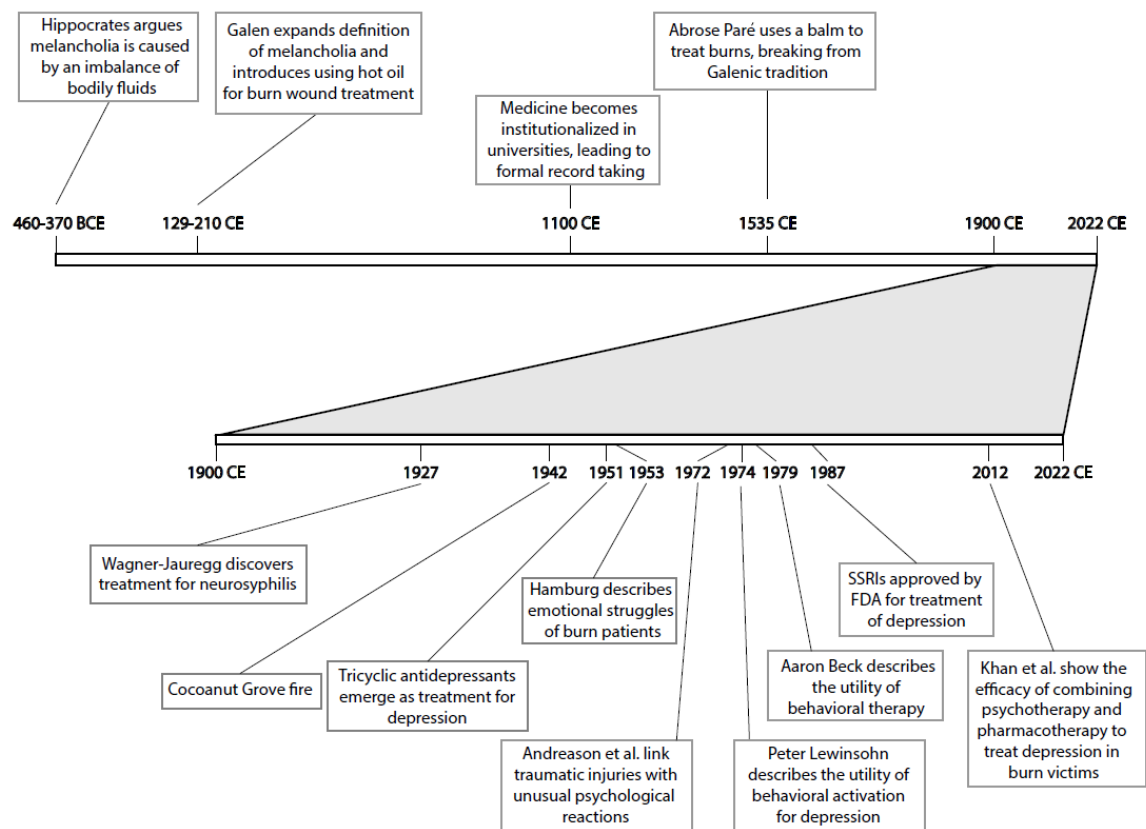

**Figure S1.** Milestones in the Management of Depression and Stress Reactions in Burn Care .

Supplement: Supplementary file 1 [file medicina-58-01395-s001.zip › medicina-1858580-Supplementary.pdf]
